# Histopathologic Progression and Metastatic Relapse Outcomes in Small Cell Neuroendocrine Carcinomas of the Urinary Tract

**DOI:** 10.1002/cam4.70594

**Published:** 2025-01-20

**Authors:** Mohammad Jad Moussa, Georges C. Tabet, Arlene O. Siefker‐Radtke, Lianchun Xiao, Nathaniel R. Wilson, Jianjun Gao, Christopher J. Logothetis, Petros Grivas, Byron Lee, Amishi Y. Shah, Pavlos Msaouel, Roger Li, Leticia Campos Clemente, Jianping Zhao, Nizar M. Tannir, Ashish M. Kamat, Donna E. Hansel, Charles C. Guo, Matthew T. Campbell, Omar Alhalabi

**Affiliations:** ^1^ Division of Cancer Medicine, Department of Genitourinary Medical Oncology The University of Texas MD Anderson Cancer Center Houston Texas USA; ^2^ Division of Pathology and Laboratory Medicine, Department of Pathology The University of Texas MD Anderson Cancer Center Houston Texas USA; ^3^ Division of Hematology and Oncology, Department of Internal Medicine University of Michigan Ann Arbor Michigan USA; ^4^ Division of Hematology and Oncology, Department of Medicine University of Washington School of Medicine Seattle Washington USA; ^5^ Clinical Research Division Fred Hutchinson Cancer Center Seattle Washington USA; ^6^ Division of Surgery, Department of Urology The University of Texas MD Anderson Cancer Center Houston Texas USA; ^7^ Department of Genitourinary Oncology H. Lee Moffitt Cancer Center Tampa Florida USA; ^8^ Department of Translational Molecular Pathology The University of Texas MD Anderson Cancer Center Houston Texas USA

**Keywords:** bladder cancer, metastatic relapse, neuroendocrine carcinoma, prognostic factors, small cell carcinoma, surgical outcomes, urothelial carcinoma

## Abstract

**Introduction:**

Small cell neuroendocrine carcinoma of the urinary tract (SCNEC‐URO) has an inferior prognosis compared to conventional urothelial carcinoma (UC). Here, we evaluate the predictors and patterns of relapse after surgery.

**Materials and Methods:**

We identified a definitive‐surgery cohort (*n* = 224) from an institutional database of patients with cT1‐T4NxM0 SCNEC‐URO treated in 1985–2021. Histopathologic review was conducted by independent pathologists. Relapse event was the time‐to‐event outcome, and relapse probabilities were estimated using a competing risk method with cumulative incidence functions (CIFs). Fine‐Gray distribution models assessed covariate associations.

**Results:**

Most patients (161, 71.9%) received neoadjuvant chemotherapy (neoCTX). Ninety two (41%) patients had relapse with 77 (83.7%) having distant organs as first metastatic sites, including 10 (10.9%) with exclusive central nervous system (CNS) metastases, mostly (9/10) within 1 year of surgery. Patients with pathologic complete response (pCR) after neoCTx had the lowest 5‐year CIF (16.5% [95% CI 9.3%–25.6%]). Patients with remaining exclusively small cell (SC) histology had the highest CIF (85.7% [95% CI 46.6–96.9]). Patients with eradicated SCNEC but remaining UC components had an intermediate‐risk CIF (32.5% [95% CI 18.6–47.2]). Multivariable analysis adjusting for neoCTx, clinical stage at diagnosis (T3/4, N0/N+ vs. T1/T2, N0), and pathologic stage (pN+ vs. pN0) demonstrated that any SCNEC histology at resection (vs. pCR) was associated with relapse risk (hazard ratio = 3.69 [95% CI 1.91–7.13], *p* = 0.0001).

**Conclusions:**

SCNEC‐URO is a systemic disease with high risk of distant relapse including CNS. Our findings highlight unmet needs for neoadjuvant/adjuvant approaches targeting the rare SCNEC subtype and suggest adding CNS surveillance within the first year after definitive surgery to high‐risk patients.

**Précis (Condensed Abstract):**

Alongside neoadjuvant chemotherapy and cancer stage, histology at resection strongly impacts relapse risk in small cell neuroendocrine carcinomas of the urinary tract. The incidence of brain metastasis is notably higher than in “traditional” urothelial cancer within the first year after surgery, especially if small cell cancer persists, thus necessitating close neurological monitoring during this period.

## 1 Introduction

Highly aggressive and predominantly metastatic, small cell neuroendocrine carcinoma of the urinary tract (SCNEC‐URO) has limited therapeutic options, with only about 10%–40% of patients surviving beyond 5 years, compared to 75%–80% for urothelial carcinoma (UC) [1, 2]. Despite the chemosensitive nature of the disease, many patients experience relapse, with a median overall survival (OS) in the metastatic setting of 5–13.3 months [3, 4, 5].

Historically, metastatic SCNEC‐URO is challenging to treat, despite our best reported regimens; for example, a small series using ifosfamide plus doxorubicin and etoposide plus cisplatin (IA/EP) demonstrated a high initial overall response rate (11/12, 91.7%) but limited survival benefit (median OS: 13.3 months) [3]. Because the rarity of the disease precludes large prospective randomized studies, management is tentatively extrapolated from SCNEC of the lung (SCLC), despite differences in histological features [6, 7]. SCNEC‐URO is predominantly (in 60%–70% of cases) found admixed with other morphologic subtypes, mainly conventional UC—but less frequently, adenocarcinoma, micropapillary, sarcomatoid, and squamous histologies—whereas combinations of tumor types are seen only in 5%–28% of all SCLC cases [8, 9, 10]. These mixed patterns are explained by the totipotent stem‐cell theory, showing concordance in loss of heterozygosity between co‐existing SCNEC and UC clones, as well as a largely similar profile of mutations and amplifications between SCLC and non‐SCLC components [8, 11]. With no consensus about the terminology of these mixed histological types, the genitourinary pathology community usually prefers to annotate the percentages of the NEC component and the non‐NEC component [12].

Although “mixed” and “pure” SCNEC have been described at localized disease, their distribution at surgical resection or relapse remains largely unknown. Understanding the biological behavior of the mixed components is essential to predicting the risk of relapse, patterns of relapse and ‘customizing’ systemic treatments to reduce those risks [9]. Recent advancements in management of metastatic UC with antibody‐drug conjugates (ADC), targeting Nectin‐4, and human epidermal growth factor receptor 2 (HER2) [13, 14, 15], leave an unanswered question of the best strategy to treat relapsed SCNEC with these agents versus other agents that have activity in SCLC [16].

Here, we aimed to assess predictors and patterns of relapse in SCNEC‐URO by conducting an original clinicopathologic study of histological subtypes from localized disease/transurethral resection (TUR) to definitive surgical resection to metastatic disease, then by analyzing clinical outcomes associated with metastatic relapse. We hypothesized that distinct histologies at definitive surgery are associated with varying likelihood of metastatic relapse in surgically resectable SCNEC‐URO.

## 2 Patients and Methods

### 2.1 Patient Population and Study Design

Data were extracted from an institutional cohort of 403 patients with SCNEC‐URO treated at MD Anderson Cancer Center (Houston, TX, USA) between November 1985 and June 2021. We excluded 95 patients with ‘synchronous’ metastasis, defined as metastasis at initial diagnosis or confirmed by imaging within 90 days of diagnosis. Thus, a pool of 308 patients with cT1‐T4NxM0 surgically operable SCNEC‐URO were initially included. Two cohorts (A and B) were selected for analysis (Figure S1). For the study of progression from TUR to surgical resection and metastatic relapse outcomes, we identified Cohort A (*n* = 224), patients who had undergone surgical resection for localized or locally advanced disease. Sites of relapse were categorized as either “distant” or “local”. A “local” relapse refers to a recurrence within the surgical bed or the nearest regional nodal basin, as opposed to “distant” sites such as anatomically farther lymph nodes and visceral organs. For the study of histology at the time of metastasis, we analyzed Cohort B (*n* = 61), patients who underwent metastatic‐site biopsies for relapse or “metachronous” metastasis. Cohort B included 51 patients from Cohort A and 10 patients who had not undergone resection. The study was approved by MD Anderson's Institutional Review Board (IRB) (Protocol no. PA16‐0736).

### 2.2 Histological Definitions

Histological annotations were abstracted from pathology reports of specimens collected at three timepoints: TUR, definitive surgery, and diagnosis of metastatic disease. The relative amount of SCNEC in the tumor was calculated by comparing the SCNEC area over the total tumor area on the available slides. The tumors were divided into the 3 following groups—minority SCNEC (SCNEC comprising < 50% of the tumor), predominant SCNEC (SCNEC comprising ≥ 50% of the tumoral specimen), and SCNEC only. Additionally, at surgery and metastatic disease, specimens with eradicated SCNEC components were labeled “non‐SCNEC.” For regrouping purposes, the umbrella term of “any SCNEC” encompassed all the tumors with SCNEC, including minority SCNEC, predominant SCNEC, and SCNEC only. Pathologic complete response (pCR) from neoadjuvant chemotherapy (neoCTx) or TUR, termed ‘all pCR,’ was defined as pT0N0/pTisN0 or ypT0N0/ypTisN0 at surgical resection. A second assessment of the histological annotations by our pathologists (G.C.T. and C.C.G.) ensured readout consistency given the long timespan of the cohort.

### 2.3 Endpoints

Our primary endpoint was to describe the patterns and first sites of relapse. Our secondary endpoint was to assess the prognostic significance of different histological outcomes at resection, along with associated clinicopathologic variables, in determining postsurgical relapse, based on the evolution of SCNEC histologies between TUR and definitive surgery (Cohort A). Our exploratory endpoint was to characterize SCNEC histologies at metastatic disease for patients with metastatic‐site biopsies (Cohort B).

### 2.4 Statistical Analysis

Continuous variables were compared using the Wilcoxon rank sum test, while categorical variables were compared using Fisher's exact test. Relapse events were analyzed as time‐to‐event (TTE) outcomes, applying a competing‐risk approach including death without known relapse. Cumulative incidence functions (CIFs) for relapse were estimated using non‐parametric methods, with subgroup comparisons conducted using the Fine‐Gray test. Fine‐Gray subdistribution models were used to evaluate the association of time to relapse with covariates.

To test our hypothesis while minimizing bias, we designed a directed acyclic graph (DAG) to codify our prespecified model [17] (Figure S2). Accordingly, our multivariable Cox regression model for relapse outcomes was adjusted for DAG confounders, stage at resection and neoCTx. Stage at diagnosis was a prognostic variable, increasing the precision of the total‐effect estimate of histology at resection on relapse outcomes [18]. Kaplan–Meier plots were used to visualize survival curves, with median OS times and log‐rank *p* values obtained with GraphPad Prism version 9.2.0 software (San Diego, CA, USA).

## 3 Results

### 3.1 Baseline Characteristics of Patients: Cohort A

Clinical characteristics of Cohort A (*n* = 224) are summarized in Table 1. The majority of patients had mixed SCNEC histology at diagnosis, in both patients who had and had not received neoCTx (120, 74.5% and 45, 71.4%, respectively), including predominant SCNEC (88, 54.7% and 28, 44.4%) and minority SCNEC (32, 19.9% and 17, 27%). Most patients who underwent neoCTx (161, 71.9%) received our established regimens targeting the SCNEC component: etoposide plus cisplatin (EP) or alternating regimens of ifosfamide plus doxorubicin (adriamcyin) and etoposide plus cisplatin (IA/EP) (131/161, 81.4%). Patients who did not receive neoCTx (63, 28.1%) underwent upfront surgical resection.

**TABLE 1:** Baseline characteristics of cohort A (*n* = 224).

Covariate		NeoCTx		Fisher's exact test *p*
		N (*n* = 63)	Y (*n* = 161)	
Sex	Female	16 (25.4%)	23 (14.3%)	0.0762
	Male	47 (74.6%)	138 (85.7%)	
Race/ethnicity	White or Caucasian	56 (88.9%)	137 (85.1%)	0.9848
	American Indian or Alaskan	0 (0%)	1 (0.6%)	
	Asian	1 (1.6%)	3 (1.9%)	
	Black or African American	1 (1.6%)	4 (2.5%)	
	Hispanic or Latino	5 (7.9%)	12 (7.5%)	
	Other	0 (0%)	3 (1.9%)	
	Unknown	0 (0%)	1 (0.6%)	
Clinical stage at diagnosis	T1 N0	5 (7.9%)	17 (10.6%)	0.3824
	T2 N0	39 (61.9%)	98 (60.9%)	
	T3/4 N0	12 (19%)	25 (15.5%)	
	Tx N0	4 (6.3%)	4 (2.5%)	
	Any N+	3 (4.8%)	17 (10.6%)	
Date of initial identification of SCNEC histology	1985–2008	46 (73%)	49 (30.4%)	< 0.0001
	2009–2021	17 (27%)	112 (69.6%)	
Primary cancer	Bladder	55 (87.3%)	155 (96.3%)	0.037
	Renal pelvis or ureter	5 (7.9%)	4 (2.5%)	
	Urethra	2 (3.2%)	2 (1.2%)	
	Urachus	1 (1.6%)	0 (0%)	
Type of neoCTx	SCNEC‐based regimens^a^	—	131 (81.4%)	—
	UC‐based regimens^b^	—	30 (18.6%)	
Histology at localized disease	SCNEC only	18 (28.6%)	41 (25.5%)	0.3433
	Predominant SCNEC	28 (44.4%)	88 (54.7%)	
	Minority SCNEC	17 (27%)	32 (19.9%)	
Co‐presence of CIS at localized disease	Y	36 (57.1%)	105 (65.2%)	0.2835
	N	27 (42.9%)	56 (34.8%)	
Histology at resection^c^	Any SCNEC (*n* = 89)	48 (76.2%)	41 (25.5%)	< 0.0001
	Minority SCNEC (*n* = 16)	12 (19%)	4 (2.5%)	
	Predominant SCNEC (*n* = 59)	32 (50.8%)	27 (16.8%)	
	SCNEC only (*n* = 14)	4 (6.3%)	10 (6.2%)	
	Non‐SCNEC (*n* = 43)	8 (12.7%)	35 (21.7%)	
	All pCR (*n* = 92)	7 (11.1%)	85 (52.8%)	
	pCR after TUR (*n* = 7)	7 (11.1%)	0 (0%)	
	pCR after neoCTx (*n* = 85)	0 (0%)	85 (52.8%)	
Pathologic stage at resection	pT0 N0	3 (4.8%)	57 (35.4%)	< 0.0001
	pTis N0	5 (7.9%)	28 (17.4%)	
	pTa N0	0 (0%)	2 (1.2%)	
	pT1 N0	4 (6.3%)	9 (5.6%)	
	T2 or greater, N0	38 (60.3%)	45 (28%)	
	Any N+	13 (20.6%)	20 (12.4%)	
ECOG performance status at diagnosis	0	10 (15.9%)	80 (49.7%)	< 0.0001
	1	37 (58.7%)	73 (45.3%)	
	≥ 2 (2–4)	2 (3.2%)	4 (2.5%)	
	N/a	14 (22.2%)	4 (2.5%)	
Adjuvant chemotherapy	Y	26 (41.3%)	8 (5%)	< 0.0001
	N	24 (38.1%)	148 (91.9%)	
	N/a	13 (20.6%)	5 (3.1%)	
Metastatic relapse outcome	Later	40 (63.5%)	52 (32.3%)	0.0001
	Never	22 (34.9%)	102 (63.4%)	
	N/a	1 (1.6%)	7 (4.3%)	

Abbreviations: CIS: carcinoma in situ; ECOG: Eastern Cooperative Oncology Group; NeoCTx: neoadjuvant chemotherapy; pCR: pathologic complete response; SCNEC: small cell neuroendocrine carcinoma; TUR: transurethral resection; UC: urothelial carcinoma.

^a^ Etoposide plus cisplatin (EP): *n* = 50; ifosfamide plus doxorubicin (adriamycin), alternating with etoposide plus cisplatin (IA/EP): *n* = 81.

^b^ Dose‐dense methotrexate, vinblastine, doxorubicin, and cisplatin (ddMVAC): *n* = 5; gemcitabine plus cisplatin (Gem/Cis): *n* = 3; paclitaxel, methotrexate, cisplatin (TMP): *n* = 3; other regimens: *n* = 19.

^c^ Types of surgical resection were radical cystoprostatectomy (159, 71%), radical cystectomy (31, 13.8%), partial cystectomy (17, 7.6%), radical nephrectomy/nephroureterectomy (8, 3.6%), radical cystourethrectomy (5, 2.2%), radical cystectomy/cystoprostatectomy with nephroureterectomy (2, 0.9%), subtotal ureterectomy (1, 0.4%), and bladder diverticulectomy (1, 0.4%).

**FIGURE 1:**
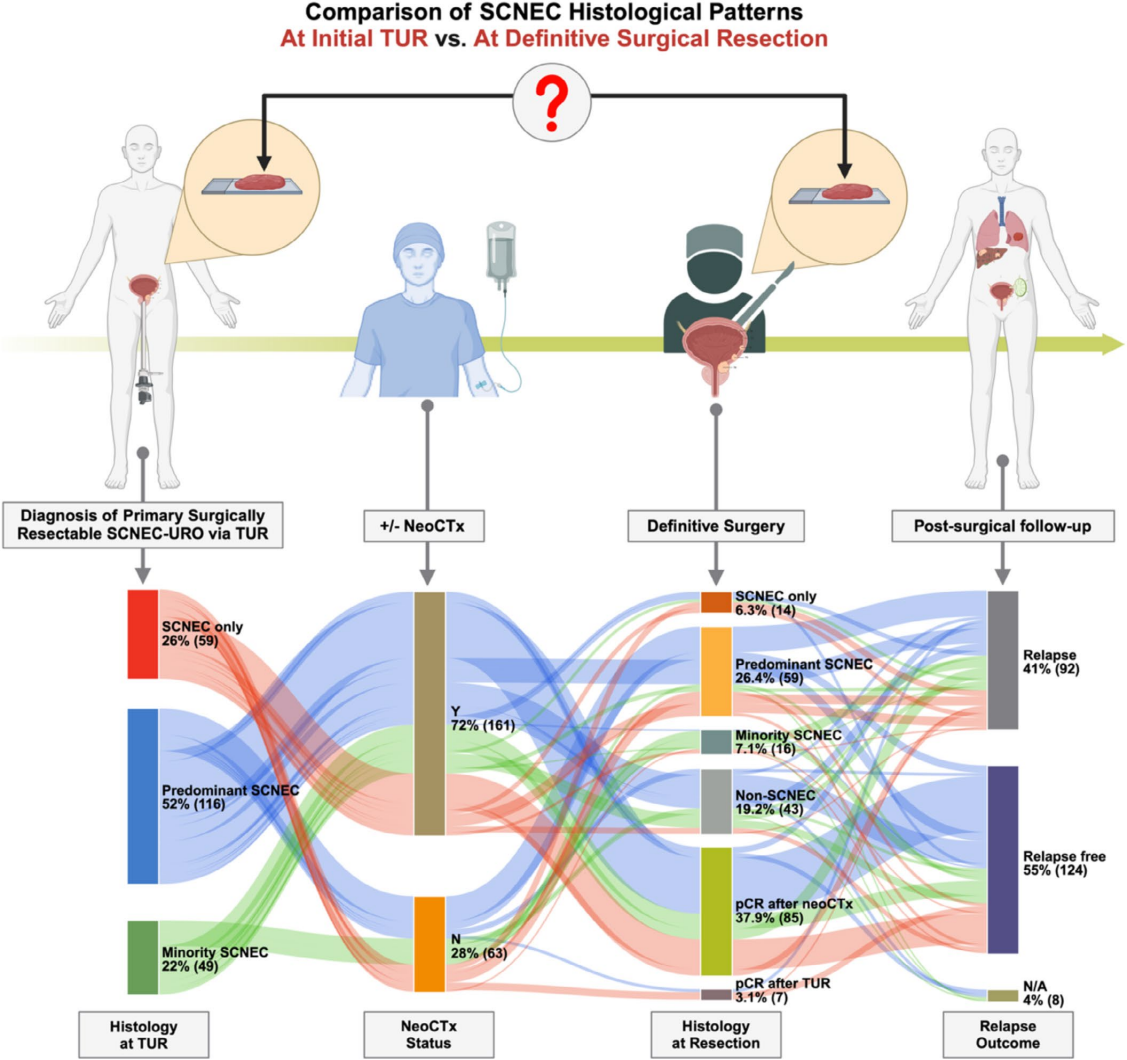
Sankey diagram showing the therapeutic evolution of Cohort A (*n* = 224) at four timepoints: Their original histology at transurethral resection, neoadjuvant chemotherapy status, histology at surgical resection and follow‐up of their metastatic status after surgery. N/A: not assessed (lost to follow up), N: no, NeoCTX: neoadjuvant chemotherapy; pCR: pathologic complete response; SCNEC‐URO: small cell neuroendocrine carcinomas of the urinary tract; TUR: transurethral resection, Y: yes.

To assess the prognostic significance of histological subtype at definitive surgery, we correlated disease relapse status with select clinical variables, as depicted in Figure 1. Following definitive surgery, patients without neoCTx experienced almost twice the incidence of relapse compared to those who had received neoCTx (63.5% vs. 32.3%, respectively). Among patients with no relapse (124, 55%), the majority achieved pCR after neoCTx (*n* = 67); conversely, among patients with relapse (92, 41%), most had predominant SCNEC at resection (*n* = 41).

### 3.2 Patterns of Relapse in Cohort A

We studied the first documented relapse patterns in patients with failed events (*n* = 92) (Figure 3). The majority of patients had distant recurrence (77, 83.7%). Relapses mostly included the liver (24, 26.1%), lung (18, 19.6%) and bone (17, 18.5%). A minority of patients had local recurrence only (12, 13%), as the iliac, inguinal or generally pelvic nodes were mostly involved, and only 5 patients received adjuvant radiation therapy for disease control. Notably, among all patients with relapse, 10 (10.9%) had an exclusive first relapse in the brain, without concomitant extracranial spread, with a median relapse time from definitive surgery of 6.8 months [IQR: 1.8–11.6] (Table S2). Of these, 7 patients had residual disease at surgery (5 with remaining SCNEC, 2 with UC) and 3 patients achieved pCR after neoCTx.

### 3.3 Risk Factors for Relapse of Disease

Time‐to‐event (TTE) outcomes were available for 216 patients; 8 patients lacked precise documentation of the timing of relapse. Estimates of CIF for metastatic relapse (Table 2) showed that 92 patients experienced metastasis (failure events), 70 remained alive without relapse (censored events), and 54 died without documented metastatic relapse (competing events). The estimated 5‐year CIF of metastasis for all patients was 41.9% (95% confidence interval [CI] 35.2%–48.5%).

**TABLE 2:** Estimated 5‐year cumulative incidence rates of metastasis for subgroups of patients by each of variables, as well as *p*‐values from Gray's test to evaluate the significance in difference of cumulative incidence function between subgroups of patients.

	Summary of failure outcomes (*n* = 216)				Cumulative incidence function (CIF) estimates				
Parameter	Failed events	Competing events	Censored	Total	Time point (months) to estimate CIF	Cumulative incidence (%)	95% CI		Gray's test *p*
Histology at resection									
Any SCNEC	61	15	11	87	60	69.2	58.1	77.9	< 0.0001
Minority SCNEC	8	3	4	15	60	53.3	24.9	75.3	
Predominant SCNEC	41	12	5	58	60	69	55	79.4	
SCNEC only	12	0	2	14	60	85.7	46.6	96.9	
Non‐SCNEC	13	12	15	40	60	32.5	18.6	47.2	
All pCR	18	27	44	89	60	18.7	11.2	27.7	
pCR after TUR	3	2	2	7	60	42.9	6.6	76.8	
pCR after neoCTx	15	25	42	82	60	16.5	9.3	25.6	
Histology at resection (regrouped)									
No pCR after neoCTx	77	29	28	134	60	56.8	47.9	64.8	< 0.0001
pCR after neoCTx	15	25	42	82	60	16.5	9.3	25.6	
Pathologic stage at resection									
pT0 N0	8	19	31	58	60	14.4	6.7	25	< 0.0001
pTis N0	10	9	13	32	60	25	11.6	41	
pTa N0	1	1	0	2	60	NE	NE	NE	
pT1 N0	3	2	7	12	60	25	5.4	51.8	
T2 or greater, N0	43	21	17	81	60	51.9	40.4	62.2	
Any N+	27	2	2	31	60	NE	NE	NE	
Pathologic stage at resection (regrouped)									
pN+ or greater	27	2	2	31	50	88.7	64.3	96.8	< 0.0001
pN0 group	65	52	68	185	50	34.2	27.3	41.1	
NeoCTx									
No	40	15	7	62	60	63.1	49.5	74	0.0002
Yes	52	39	63	154	60	33.2	25.7	40.7	
Primary cancer									
Bladder	84	52	67	203	60	40.6	33.7	47.4	0.0913
Renal pelvis or ureter	6	2	1	9	60	NE	NA	NA	
Urethra	1	0	2	3	60	33.3	0.1	83.2	
Urachus	1	0	0	1	60	NE	NA	NA	

Abbreviations: neoCTx: neoadjuvant chemotherapy; pCR: Pathologic complete response; SCNEC: Small cell neuroendocrine carcinomas.

CIF estimates significantly differed by histological subtype at resection (Gray's test, *p* < 0.0001). Patients achieving pCR with neoCTx had the most favorable outcomes, with the lowest 5‐year CIF for relapse (16.5% [95% CI 9.3%–25.6%]). Patients achieving pCR with TUR had a higher CIF of 42.9% (95% CI 6.6%–76.8%). In comparison, patients with any remaining SCNEC component had the least favorable outcomes, with an exclusive SC histology (SCNEC only) showing the highest CIF (85.7% [95% CI 46.6%–96.9%]). Intermediate CIF estimates were noted for patients with exclusive remaining UC (non‐SCNEC: 32.5% [95% I 18.6%–47.2%]) and patients with varying degrees of SCNEC presence—decreasing from SCNEC only (85.7% [95% CI 46.6%–96.9%]) to predominant SCNEC (69% [95% CI 55%–79.4%]) to minority SCNEC/predominant UC (53.3% [95% CI 24.9%–75.3%]).

Staging at resection also showed different CIFs (Gray's test, *p* < 0.0001), increasing gradually with higher tumor stages (pT0N0: 14.4% [95% CI 6.7%–25%]; pT1N0: 25% [95% CI 5.4%–51.8%]; and pT2 or greater, N0: 51.9% [95% CI 40.4%–62.2%]). No estimated 5‐year CIF was obtained for patients staged as node positive (pN+) because all either relapsed (27/31), died (2/31), or were lost to follow‐up (2/31) within 5 years. We therefore estimated CIF at an earlier time point of 50 months and found a relapse probability of 88.7% (95% CI 64.3%–96.8%) in the N+ group, compared to 34% (95% CI 27.3%–41.1%) for all patients regrouped as N0, regardless of T stage.

**FIGURE 2:**
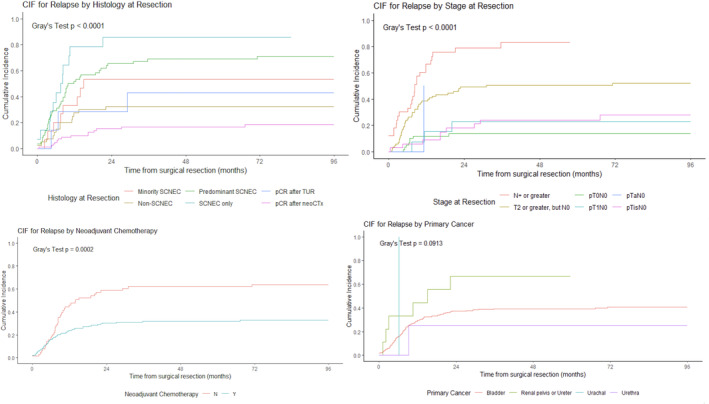
Cumulative incidence function curves for metastasis by histology at resection, neoadjuvant chemotherapy status, primary cancer type, and stage at resection. CIF: cumulative incidence function; N: no, neoCTx: neoadjuvant chemotherapy; pCR: pathologic complete response; SCNEC: small cell neuroendocrine carcinoma; Y: yes.

The established survival benefit of neoCTx was demonstrated by a significantly lower 5‐year CIF of metastatic relapse in the neoCTx group compared to the group without neoCTx (33.2% [95% CI 25.7%–40.7%] vs. 63.1% [95% CI 49.5%–74%], *p* = 0.0002). The CIF curves according to histological subtype at resection, primary cancer type, and stage at resection are shown in Figure 2.

**FIGURE 3:**
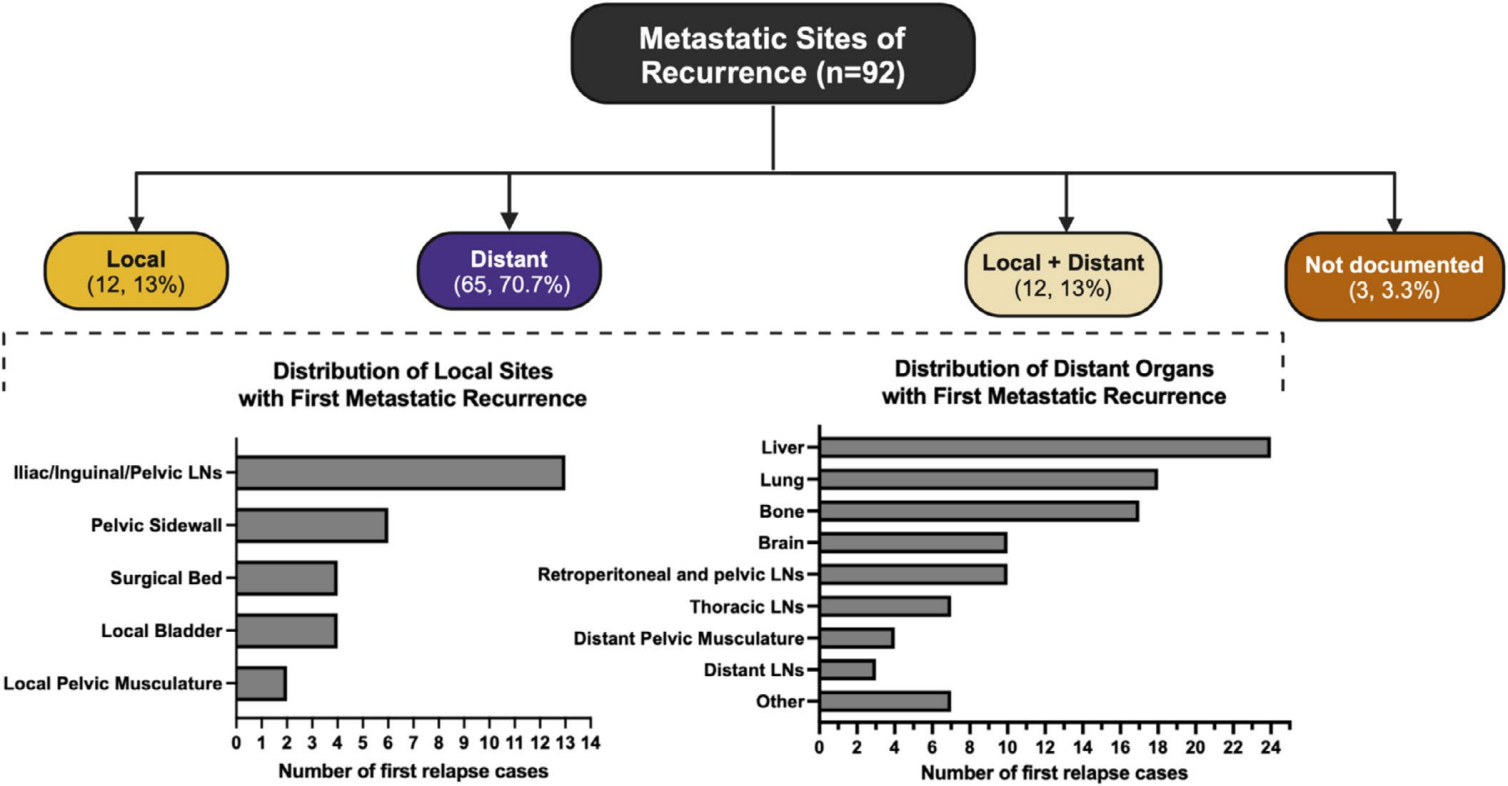
Distribution of anatomic sites among patients with metastatic recurrence in Cohort A (*n* = 92). LNs: lymph nodes.

Univariable analysis indicated that pathologic criteria of histology and stage at resection, as well as neoCTx, were associated with postsurgical relapse. Patients with pCR had significantly reduced relapse rates (by around three‐quarters) than did patients without pCR (*p* < 0.0001) (Table S1).

In the multivariable model (Table 3), patients with nodal disease were more than twice as likely to experience relapse than those without nodal involvement (pN0), regardless of pT staging (hazard ratio [HR] 2.4 [95% CI 1.4–4], *p* = 0.0009). Higher‐risk pathologic stages (T3/T4, N0/N+) were also associated with increased relapse risk compared to lower stages (T1/T2, N0) (HR 1.6 [95% CI 1.1–2.5], *p* = 0.02). Interestingly, relapse risk was highest for specimens showing any SCNEC compared to those showing eradication of both SC and UC components in the all‐pCR group (HR 3.7 [95% CI 1.9–7.1], *p* = 0.0001).

**TABLE 3:** Multivariable analysis for metastatic relapse.

Parameter		HR	95% CI		*p*
Histology at resection	Any SCNEC versus All pCR	3.688	1.908	7.128	0.0001
	Non‐SCNEC versus All pCR	1.402	0.687	2.861	0.3539
Clinical stage at diagnosis	T3/T4, N0/N+ versus T1‐T2N0	1.634	1.065	2.508	0.0246
Pathologic stage at resection	pN+ versus pN0 group	2.389	1.427	4	0.0009
NeoCTx status	No versus Yes	1.068	0.643	1.775	0.7988

Abbreviations: CI: confidence interval; HR: hazard ratio; neoCTx: neoadjuvant chemotherapy; pCR: pathologic complete response; SCNEC: small cell neuroendocrine carcinoma.

### 3.4 Histological Subtypes at Metastatic Disease for Relapsing Tumors: Cohort B

Cohort B (*n* = 61) was established to track the histopathological progression in biospecimens from patients with relapsing disease. Most metastatic biopsy locations were nodal or hepatic (Figure S3). Roughly half of patients had neoCTx (31, 50.8%), including NEC‐targeted regimens (21/31, 67.7%), and most (50, 82%) had surgical resection (Table S3).

At TUR, approximately three‐quarters of histologies were mixed patterns, with predominant SCNEC in 30 patients (49%) and minority SCNEC in 17 (28%); the rest (*n* = 14, 23%) were SCNEC only. The most prevalent co‐existent pathology in mixed SCNEC was UC (38/61, 62.3%). At metastatic disease, most biopsies reflected an exclusive SCNEC component (39, 64%). Most predominant SCNEC cases at the time of TUR (21/30, 70%) were SCNEC only at the time of metastatic disease, and most SCNEC only cases at TUR (13/14, 92.8%) stayed the same, while minority SCNEC cases (*n* = 17) did not follow a clear evolution pattern at metastatic disease (Figure S4).

For all 61 patients, the median OS was 10.7 months (95% CI 7.5–13.8) (Figure S5). The median OS durations for each metastatic subtype were 12.2 months (95% CI 7.9–18.2) for the SCNEC only cases (*n* = 39), 9.02 months (95% CI 2.1–13.8) for the predominant SCNEC cases (*n* = 10), and 5.46 months (95% CI 2.4–18.5) for the non‐SCNEC cases (*n* = 10). At the time of metastatic‐site biopsy, most Cohort B patients had visceral metastasis (*n* = 50, 82%), across the three histological subtypes. A breakdown of visceral metastasis by organ and nodal‐only metastasis, is shown in Table S3.

## 4 Discussion

The current study, analyzing the largest cohort to date of patients with surgically resected SCNEC‐URO, characterized previously unrecognized relapse patterns. Our observation that 83.7% of relapses are distant in Cohort A supports the view of SCNEC‐URO as a systemic disease with early potential for visceral dissemination. The limited incidence of locoregional recurrence (13% of relapses) casts doubt on the efficacy of tailored control strategies like adjuvant radiation therapy in this disease setting. Importantly, the high incidence of isolated first metastatic relapse in the CNS (10.9%) is consistent with data of overall higher incidence for small cell histology than for traditional UC (1%) [19]. Most intracranial relapses were diagnosed in isolation within a year, with median time to relapse of 6.8 months, particularly following residual SCNEC at resection. Thus, we recommend a baseline MRI of the brain to rule out distant spread, surveillance brain imaging every 3–4 months after surgery for the first 2 years. There is an urgent need to explore the disease biology, such as through animal models of brain metastasis, to better understand molecular underpinnings and identify therapeutic targets or biomarkers [20].

Our earlier comparative effectiveness analysis demonstrated the downstaging benefits of SCNEC‐targeted neoCTx, especially IA/EP and EP [3, 21], consistent with our current findings.

NeoCTx exerted therapeutic pressure to eradicate the SCNEC component, evidenced by a considerably higher frequency of pCR (52.8%) compared to that for TUR alone (11.1%) and a lower frequency of specimens showing any remaining SCNEC component (25.5% vs. 76.2%). However, factors associated with metastatic relapse and OS remain relatively unstudied. For example, the use of pCR as a clinical surrogate of disease‐free evolution is established in UC, but not in SCNEC‐URO [22]. Here, we provide original insights on pCR after neoCTx as a factor associated with lowest CIF relapse risk. In contrast, a four‐fold increase in relapse risk (HR = 4.1 [95% CI 2.4–7.1], *p* < 0.0001) was seen in surgical specimens with residual tumor compared to those with pCR after neoCTx (Table S1).

An aggressive multimodal therapeutic strategy is needed in the presence of any SCNEC component in a TUR specimen [23]. Hence, we were interested in determining the prognostic value of SCNEC presence, in varying degrees (minority, predominant, and exclusively SCNEC), in surgical specimens. Our results show that, regardless of its total or partial contribution, a persistent SC element, when compared to pCR, is a clear factor associated with relapse (HR = 3.7 [95% CI 1.9%–7.1%], *p* < 0.0001). Interestingly, CIF relapse rates increased gradually with the increasing SC contribution amount, ranging from 53.3% (95% CI 24.9%–75.3%) for minority (< 50%) SCNEC to 69% (95% CI 55%–79.4%) for predominant SCNEC, and 85.7% (95% CI 46.6%–96.4%) for SCNEC only. Notably, relapse risk among patients who had pCR after neoCTx appeared lower than for those who had pCR after TUR (16.5% [95% CI 9.3%–25.6%] vs. 42.9% [95% CI 6.6%–76.8%]), indicating the role of controlling early microscopic metastatic disease in SCNEC.

In Cohort A, most patients received NEC regimens such as EP and IA/EP (131/161, 81.4%), which may have influenced the remaining SCNEC component [3, 21]. Even after accounting for the confounding effect of neoCTx (Figure S2), our multivariable model demonstrated that histology and stage at resection (confounding variables in our DAG) remained strongly associated with relapse risk. This aligns with findings from Memorial Sloan Kettering Cancer Center (MSKCC); in that study, patients with response (defined as <ypT2N0) to neoCTx exhibited significantly higher median OS and disease‐free survival (DFS) compared to those without response (DFS: HR = 0.24, 14.5 vs. 0.6 years, *p* < 0.001; OS: HR = 0.31, 14.5 vs. 2.5 years, *p* = 0.002) [24]. The 5‐year DFS probabilities were 76% and 27%, respectively. Comparably, we show an almost five‐fold increase in relapse risk for patients with ≥ pT2 disease compared to patients with pT0N0 (HR = 4.82 [95% CI 2.23–10.43], *p* < 0.0001). Our model adds insights into histology at resection, not previously investigated in an evolutionary context.

The survival benefit with using SCNEC‐targeted neoCTx, like IA/EP and EP, underscores the potential of achieving pCR or, at best, reducing SCNEC proportions [21, 25]. Given the increased relapse rates with residual SCNEC, there is a rationale for investigating adjuvant therapy for any remaining SCNEC. Adjuvant immunotherapy is already established in conventional UC, showing DFS benefit with nivolumab or pembrolizumab in high‐risk muscle‐invasive UC, although OS data remains immature [26, 27].

To identify the driving component behind relapse, Cohort B helped describe the histological subtypes from metastatic‐site biopsies. Most biopsies demonstrated an exclusive SCNEC component (39/61, 64%). This likely represents the rapid proliferation and early metastatic potential observed with SCNEC versus the other (mainly urothelial) components. The bleak prognosis of Cohort B (median OS: 10.66 months [95% CI 7.51–13.84]) is explained by the high prevalence of visceral metastasis at the time of biopsy (82%), including metastatic liver (27.9%) and brain (26.2%) disease, across the three SCNEC groups.

Bladder SCNEC is usually found with other histological forms of bladder cancer at diagnosis [8, 9]. This study challenges the concept of SCNEC “purity,” showing high rates of mixed histologies at diagnosis and definitive surgery, even among patients not exposed to neoCTx (44/63, 69.8%). Limitations include the retrospective, single‐center design, with associated biases, and the heterogeneity in tumor sampling for pathologic readout (biopsies vs. surgical whole‐organ blocks). We addressed this bias by having our collaborators conduct a second pathologist review (G.C.T. and C.C.G.). Changes in diagnostic and therapeutic protocols from 1985 to 2021—including specimen quality control, neoCTx decisions, surgical care, and systemic therapy—present inherent limitations. Integrated molecular and clinical approaches are needed to define precision biomarkers for effective trial design. The correlations between pCR after neoCTx and *BRCA1*/*2* variations, along with loss of function of *ERRC2*, in the MD Anderson and MSKCC cohorts, respectively, warrant further investigation in translational studies [21, 24]. Moreover, the moderate sample size (and power) for comparative analyses, as well as potential selection and confounding biases, should be taken in account.

## 5 Conclusion

SCNEC‐URO represents a systemic disease with high propensity for distant sites of relapse, especially in patients with persistent SCNEC component at surgery. Histology at definitive surgery emerged as a key factor associated with relapse, even when adjusting for clinicopathological stage. Despite the pressure of best available chemotherapy regimens (EP and IA/EP) on SCNEC components, there are clear needs for better targeted neoadjuvant and adjuvant therapies to manage this aggressive subtype with poor prognosis. Given the higher incidence of brain metastases and the brief time to recurrence, providers should include CNS surveillance within the first year after surgery for patients with high‐risk features at resection.

### 5.1 Take Home Message

In small cell neuroendocrine carcinomas of the urinary tract, often admixed with urothelial carcinoma components at diagnosis, the small cell component tends to dominate as the cancer progresses. Alongside neoadjuvant chemotherapy and cancer stage, histology at resection strongly impacts relapse risk. Particularly, brain relapses are more common than in typical urothelial cancer and usually occur within a year, especially if small cell cancer remains after surgery. This highlights the need for close and vigilant CNS surveillance in the first year after surgery.

## Author Contributions

**Mohammad Jad Moussa:** conceptualization (equal), data curation (equal), formal analysis (equal), investigation (equal), methodology (equal), visualization (equal), writing – original draft (equal). **Georges C. Tabet:** data curation (equal), investigation (equal), methodology (equal), project administration (equal), validation (equal). **Arlene O. Siefker‐Radtke:** methodology (equal), validation (equal), writing – review and editing (equal). **Lianchun Xiao:** data curation (equal), formal analysis (equal), methodology (equal), software (equal), validation (equal), visualization (equal). **Nathaniel R. Wilson:** data curation (equal), validation (equal). **Jianjun Gao:** validation (equal), writing – review and editing (equal). **Christopher J. Logothetis:** writing – review and editing (equal). **Petros Grivas:** writing – review and editing (equal). **Byron Lee:** writing – review and editing (equal). **Amishi Y. Shah:** writing – review and editing (equal). **Pavlos Msaouel:** data curation (equal), formal analysis (equal), methodology (equal), writing – review and editing (equal). **Roger Li:** writing – review and editing (equal). **Leticia Campos Clemente:** project administration (equal), writing – review and editing (equal). **Jianping Zhao:** project administration (equal), resources (equal), validation (equal), writing – review and editing (equal). **Nizar M. Tannir:** validation (equal), writing – review and editing (equal). **Ashish M. Kamat:** writing – review and editing (equal). **Donna E. Hansel:** project administration (equal), resources (equal). **Charles C. Guo:** methodology (equal), project administration (equal), resources (equal), validation (equal), writing – review and editing (equal). **Matthew T. Campbell:** funding acquisition (equal), investigation (equal), methodology (equal), supervision (equal), validation (equal), writing – review and editing (equal). **Omar Alhalabi:** conceptualization (equal), funding acquisition (equal), investigation (equal), methodology (equal), resources (equal), supervision (equal), validation (equal), visualization (equal), writing – review and editing (equal).

## Ethics Statement

The study was performed in accordance with protocol PA16‐0736, approved by the MD Anderson Cancer Center Institutional Review Board.

## Consent

The protocol is an investigational study for which consent is waived.

## Conflicts of Interest

Mohammad Jad Moussa, Georges C. Tabet, Lianchun Xiao, Nathaniel R. Wilson, Byron Lee, Jianping Zhao, Leticia Campos Clemente and Charles C. Guo report no disclosures. Arlene O. Siefker‐Radtke reports scientific advisory board fees from Abbvie, Astellas, AstraZeneca, Basilea, Bicycle Therapeutics, Bristol Myers Squibb, Genentech, G1 Therapeutics, Gilead, Ideeya Biosciences, Immunomedics, Janssen, Loxo, Merck, Mirati, Nektar Therapeutics, Seattle Genetics and Taiho. Roger Li reports research support from Predicine, Veracyte, CG Oncology, Valar labs, Merck; scientific advisory board fees from BMS, Merck, Fergene, Arquer Diagnostics, Urogen Pharma, Lucence, CG Oncology, Janssen, Thericon; clinical trial support from CG Oncology; Merck; Janssen; and honoraria—SAI MedPartners, Solstice Health Communications, Putnam Associates, and UroToday. Amishi Y. Shah reports scientific advisory board fees from Bristol Myers Squibb, Exelixis, and Pfizer; and research funding from Bristol Myers Squibb, Eisai, EMD Serono, and 4D Pharma. Ashish M. Kamat reports consultant/advisory board role for Arquer Diagnostics, Asieris, Astellas, Biological Dynamics, Bristol Myers Squibb, CG Oncology, H3 Biomedicine/Eisai, Engene, FerGene, Imagin Medical, Incyte DSMB, Janssen, Medac, Merck, Photocure, ProTara, Roche, Seattle Genetics, Sessen Bio, Theralase, TMC Innovation, US Biotest, and Urogen Inc.; clinical trial support from Adolor, Bristol Myers Squibb, FKD Industries, Heat Bio‐ logics, Janssen, Merck, Photocure, Seattle Genetics, Taris, and SWOG; laboratory research support from AIBCCR, NIH, PCORI, and SPORE; and patent with cytokine predictors of response to intravesical therapy (CyPRIT), jointly with The University of Texas MD Anderson Cancer Center. Pavlos Msaouel reports honoraria for service on scientific advisory boards for Mirati Therapeutics, Bristol Myers Squibb, and Exelixis; consulting for Axiom Healthcare Strategies; nonbranded educational programs supported by DAVA Oncology, Exelixis, and Pfizer; and research funding for clinical trials from Takeda, Bristol Myers Squibb, Mirati Therapeutics, Gateway for Cancer Research, and the University of Texas MD Anderson Cancer Center. Christopher J. Logothetis serves on advisory boards for Merck, Sharpe & Dohme, Exelixis, Bayer, and Amgen; has received honoraria from Bayer and Amgen; and has received institutional funding from Janssen, ORIC Pharmaceuticals, Novartis, and Aragon Pharmaceuticals. Nizar M. Tannir reports honoraria from Pfizer, Novartis, Bristol‐Myers Squibb, Exelixis, Nektar, Calithera Biosciences, Eisai Medical Research, Ono Pharmaceutical, Oncorena; consulting/advisory relationship with BristolMyers Squibb, Novartis, Exelixis, Epizyme, Mirati Therapeutics; and other personal fees from Pfizer, Novartis, Bristol‐Myers Squibb, Exelixis, Nektar, Calithera Biosciences, Eisai Medical Research, Ono Pharmaceutical, and Oncorena. Donna E. Hansel serves on a scientific advisory board for AstraZeneca. Jianjun Gao currently reports active consultancy for Astrazeneca Pharmaceuticals. Matthew T. Campbell reports consultancy or advisory role for Astellas, AstraZeneca, AXDev, Eisai, EMD Serono, Exelixis, Genentech, Pfizer, and SeaGen; research funding from ApricityHealth, Aravive, AstraZeneca, Exelixis, Janssen, and Pfizer/EMD Serono; nonbranded educational programs from Bristol Myers Squibb, Merck, Pfizer/EMD Serono, and Roche. Omar Alhalabi reports scientific advisory board fees from Seagen, Silverback Therapeutics, Adaptimmune, Bicycle Therapeutics and Cardinal Health; educational program speaker from Curio Science and Aptitude Health; and research funding to the institution from AstraZeneca, Ikena Oncology, Genentech, and Arcus Biosciences.

## Supporting information


**Data S1.** Supporting Information.

## Data Availability

The datasets used and/or analyzed during the current study are available from the corresponding author on reasonable request.
