# Supplementary material for: Histopathologic Progression and Metastatic Relapse Outcomes in Small Cell Neuroendocrine Carcinomas of the Urinary Tract
Source: Cancer Med. 2025 Jan 20;14(2):e70594. doi: 10.1002/cam4.70594 (PMC11744726; doi:10.1002/cam4.70594)
Supplement: Supplementary file 1 — Data S1. Supporting Information. [file CAM4-14-e70594-s001.docx]

**SUPPLEMENTARY FIGURES AND TABLES**

**SUPP. FIGURE 1.** CONSORT diagram showing the inclusion and exclusion criteria implemented to analyze our two cohorts of interest (A and B).

**
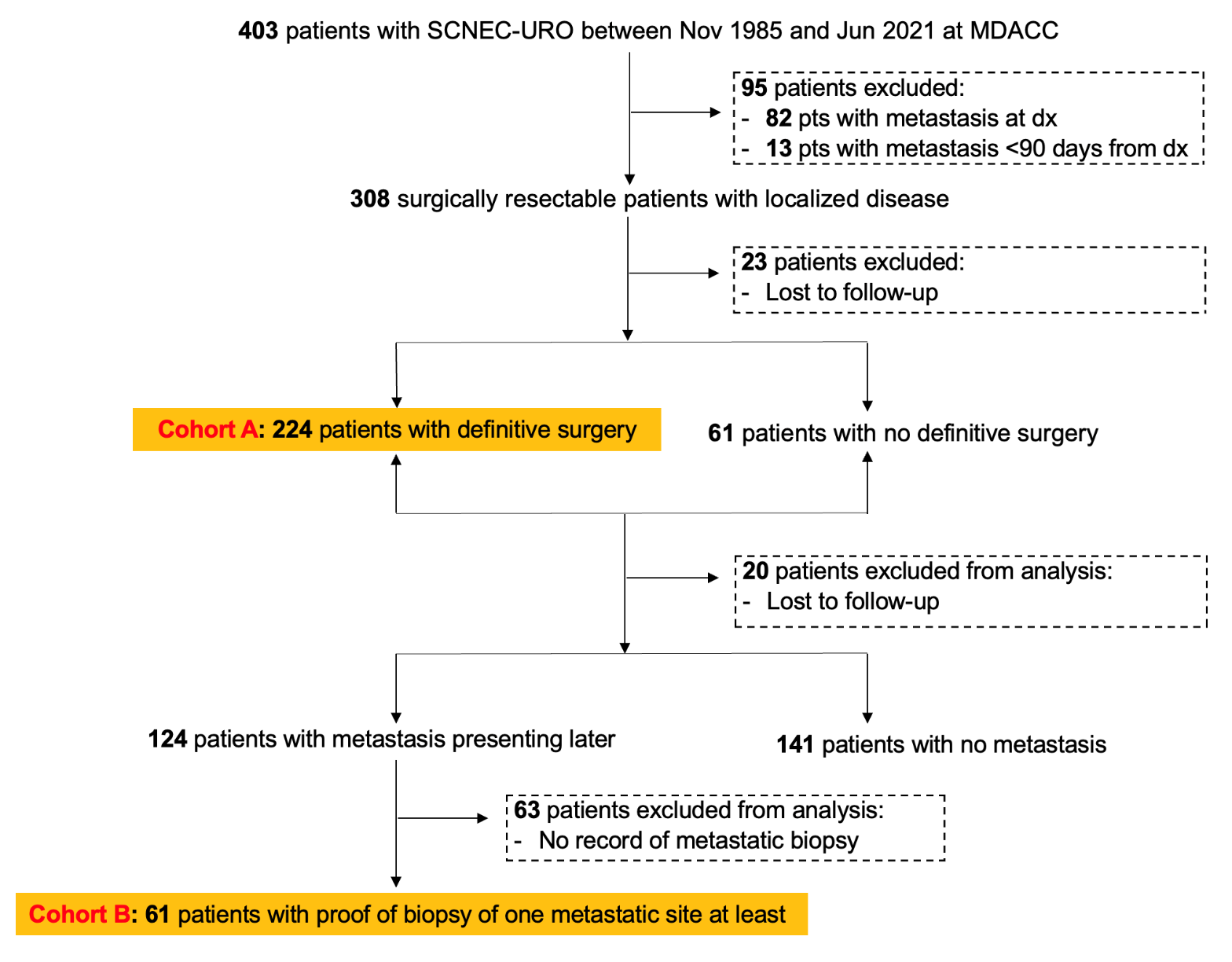
**

**SUPP FIGURE 2.** Causal effect identification between histology at resection (exposure) and risk of relapse (outcome) using a direct acyclic graph (DAG).


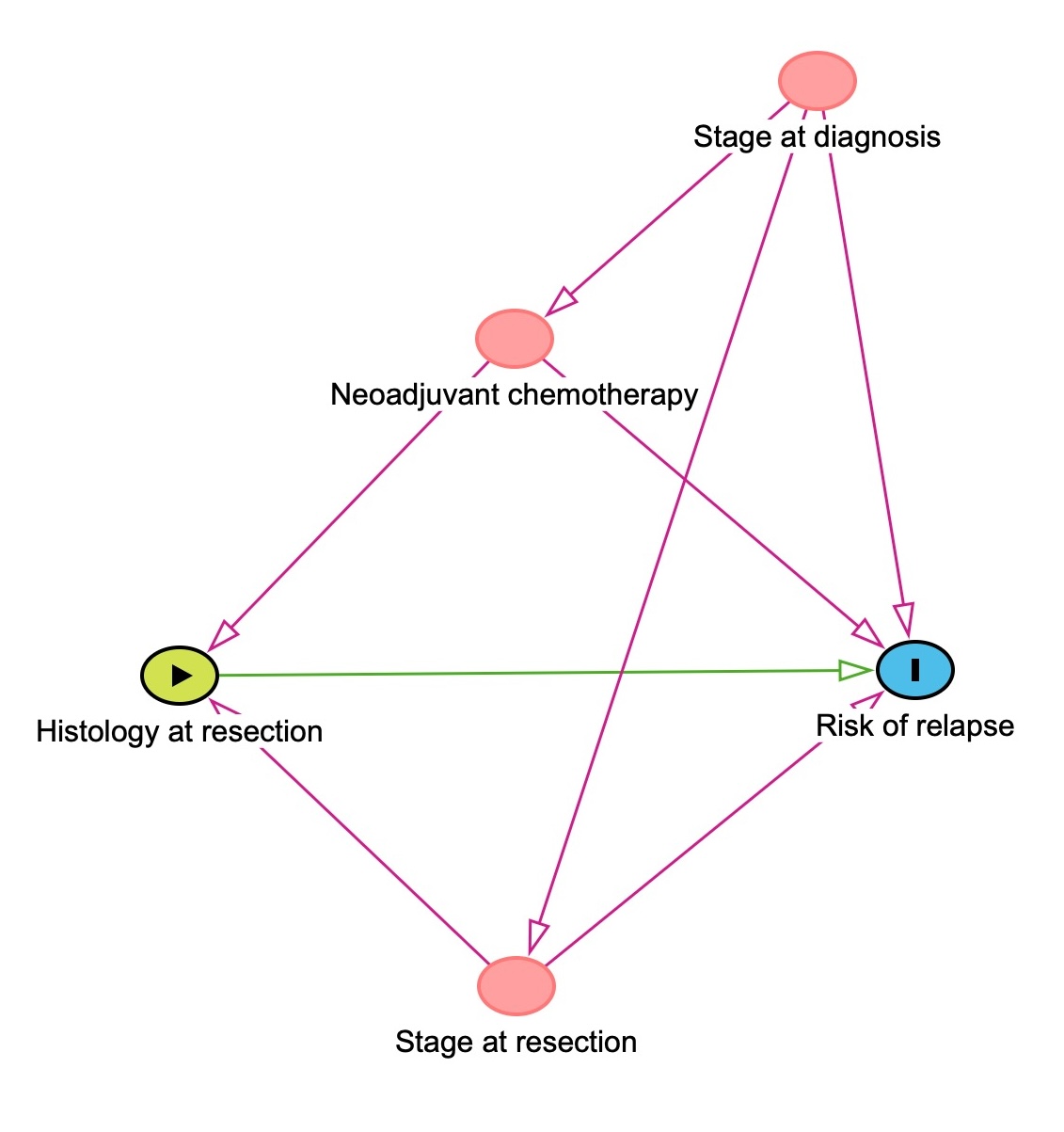


**SUPP. TABLE 1.** Univariable analysis for metastatic relapse in cohort with time-to-event outcomes (n=216).

| Parameter | | HR | 95% CI | | *p* value |
| --- | --- | --- | --- | --- | --- |
| Gender | Female (n=37) vs. Male (n=179) | 0.914 | 0.53 | 1.578 | 0.7476 |
| Age at Resection | | 0.989 | 0.971 | 1.006 | 0.2024 |
| Race | Other (n=30) vs. White or Caucasian (n=186) | 0.98 | 0.506 | 1.9 | 0.9532 |
| NeoCTx | Yes (n=154) vs. No (n=62) | 0.451 | 0.301 | 0.676 | **0.0001** |
| Primary Cancer | Non-bladder (n=13) vs. bladder (n=203) | 1.793 | 0.871 | 3.692 | 0.1132 |
| Histology at Localized Disease | Predominant SCNEC (n=111) vs.  Minority SCNEC (n=46) | 0.749 | 0.456 | 1.231 | 0.2544 |
|  | SCNEC only (n=59) vs.  Minority SCNEC (n=46) | 0.843 | 0.492 | 1.444 | 0.534 |
| ECOG PS at diagnosis | 1 (n=108) vs. 0 (n=86) | 1.377 | 0.877 | 2.162 | 0.1641 |
|  | ≥2 (n=6) vs. 0 (n=86) | 0.941 | 0.224 | 3.96 | 0.9343 |
|  | N/A (n=16) vs. 0 (n=86) | 2.296 | 1.228 | 4.294 | 0.0093 |
| Histology at Resection | All pCR (n=89) vs. Non-SCNEC (n=40) | 0.633 | 0.297 | 1.348 | 0.2355 |
|  | Any SCNEC (n=87) vs. Non-SCNEC (n=40) | 3.406 | 1.762 | 6.586 | **0.0003** |
| Histology at Resection  (Regrouped) | No pCR post neoCTx (n=134) vs.  pCR after neoCTx (n=82) | 4.135 | 2.404 | 7.112 | **<.0001** |
| Pathologic stage at Resection | pN+ or greater (n=31) vs. pT0N0 (n=58) | 11.883 | 5.283 | 26.731 | **<.0001** |
|  | pTisN0 (n=32) vs. pT0N0 (n=58) | 2.129 | 0.858 | 5.283 | 0.1031 |
|  | pTaN0 (n=2) vs. pT0N0 (n=58) | 3.963 | 0.598 | 26.252 | 0.1534 |
|  | pT1N0 (n=12) vs. pT0N0 (n=58) | 1.753 | 0.478 | 6.433 | 0.3975 |
|  | pT2 or greater, N0 (n=81) vs. pT0N0 (n=58) | 4.824 | 2.23 | 10.435 | **<.0001** |
| Pathologic stage at Resection  (Regrouped) | pN+ or greater (n=31) vs.  pN0 group (n=185) | 4.262 | 2.756 | 6.59 | **<.0001** |

HR: Hazard Ratio, CI: Confidence Interval, NeoCTx: Neoadjuvant chemotherapy; SCNEC: Small cell neuroendocrine carcinoma; ECOG PS: Eastern Cooperative Oncology Group Performance Status; pCR: Pathologic complete response.

**SUPP. TABLE 2.** Distribution of brain metastasis (n=10) in **Cohort A**. *Abbreviations*: cTNM: Clinical stage groups (Tumor, Node, Metastasis), pTNM: Pathological stage groups (Tumor, Nodes, Metastasis), mo: months, SCNEC: Small cell neuroendocrine carcinoma, pCR: Pathologic complete response, neoCTx: Neoadjuvant chemotherapy, dx: diagnosis, mets: metastasis, 1^st^: First, NE: Non-evaluable.

| Case | Brain mets  anatomic location | Presentation  of brain mets | cTNM  at original  dx | pTNM at  resection | Morphology at  resection | Time from  resection to  1^st^ relapse in  brain (mo) | Time from  brain mets  to other distant  site of  relapse (mo)  [Site] | Overall survival  from  brain mets (mo) |
| --- | --- | --- | --- | --- | --- | --- | --- | --- |
| **#1** | Right frontal lobe | Change in mental status  and ataxia (**Acute**) | cT2 N0 M0 | pT2 N0 M0 | Predominant SCNEC | **20.9** | **NE*** | **1.8** |
| **#2** | Both cerebral  hemispheres + Cerebellum | Seizure (**Acute**) | cT3b N0 M0 | pT0 N0 M0 | pCR after neoCTx | **4.6** | **NE*** | **4.7** |
| **#3** | Right cerebellum +  Left parietal lobe +  Interventricular region | On Brain MRI  (**Surveillance**) | cT4 N1 M0 | pT3 N0 M0 | Non-SCNEC  (Sarcomatous  carcinoma) | **1.3** | **7.9 mo**  **[Peritoneum]** | **21.2** |
| **#4** | Right occipital convexity | Change in mental status  and ataxia (**Acute**) | cT2 N0 M0 | pTis N0 M0 | pCR after neoCTx | **0.5** | **NE*** | **2.3** |
| **#5** | Left parietal lobe | Seizure (**Acute**) | cT1 N0 M0 | pT3 N0 M0 | SCNEC only | **5.2** | **NE*** | **8.8** |
| **#6** | Left temporal and  frontal lobes | On Brain MRI  (**Surveillance**) | cT2 N1 M0 | pT1 N0 M0 | Non-SCNEC | **11.4** | **NE*** | **4.3** |
| **#7** | Left temporal lobe | On Brain MRI  (**Surveillance**) | cT2 N0 M0 | pT3 N1 M0 | SCNEC only | **8.3** | **1 mo**  **[Liver]** | **22.9** |
| **#8** | Left supratentorial region | On Brain MRI  (**Surveillance**) | cT2 N0 M0 | pT3b N0 M0 | SCNEC only | **2.0** | **7.9 mo**  **[Pelvis]** | **10.8** |
| **#9** | Cerebellum | On Brain MRI  (**Surveillance**) | cT2 N0 M0 | pTis N0 M0 | pCR after neoCTx | **10.9** | **2 mo**  **[Lung]** | **2.7** |
| **#10** | Left temporal lobe | Word finding difficulty  and amnesia (**Subchronic**) | cT2 N0 M0 | pT2 N1 M0 | SCNEC only | **12.0** | **NE*** | **34.8** |

*No documented distant sites of metastasis after first recurrence in the brain until death, i.e. exclusive progression of brain metastasis until death.

**SUPP. TABLE 3.** Baseline characteristics of **Cohort B (n=61).** *Abbreviations*: IQR: Interquartile range, SCNEC: Small cell neuroendocrine carcinoma, ECOG PS: Eastern Cooperative Oncology Group Performance Status; NeoCTx: Neoadjuvant chemotherapy; CTx: Chemotherapy.

| Variable | Category | Measure |
| --- | --- | --- |
| Age at small cell diagnosis, median [IQR] | -- | 64 [57 – 71] |
| Sex, n (%) | Female | 8 (13.1%) |
|  | Male | 53 (86.9%) |
| Race, n (%) | White | 52 (85.2%) |
|  | Black | 3 (4.9%) |
|  | Other | 1 (1.6%) |
| Ethnicity, n (%) | Hispanic | 4 (6.6%) |
|  | Non-Hispanic | 57 (93.4%) |
| Stage at diagnosis, n (%) | T1N0 | 7 (11.5%) |
|  | T2N0 | 37 (60.6%) |
|  | T3/4N0 | 9 (14.8%) |
|  | TxN0 | 1 (1.6%) |
|  | N+ | 7 (11.5%) |
| Primary tumor location, n (%) | Bladder | 56 (91.8%) |
|  | Renal Pelvis | 3 (5%) |
|  | Urethra | 1 (1.6%) |
|  | Urachus | 1 (1.6%) |
| Histology at diagnosis, n (%) | Minority SCNEC | 17 (27.9%) |
|  | Predominant SCNEC | 30 (49.2%) |
|  | SCNEC Only | 14 (22.9%) |
| ECOG PS, n (%) | 0 | 15 (29.4%) |
|  | 1 | 30 (58.8%) |
|  | N/A | 16 (26.2%) |
| Surgical intervention at definitive treatment, n (%) | Radical cystectomy | 40 (65.6%) |
|  | Partial cystectomy | 7 (9.8%) |
|  | Other* | 3 (4.9%) |
|  | No resection | 11 (18%) |
| NeoCTx, n (%) | EP | **10 (16.4%)** |
|  | IA/EP | 11 (18%) |
|  | ddMVAC | 1 (1.6%) |
|  | Gem/Cis | 2 (3.3%) |
|  | Other**^†^** | 5 (8.2%) |
|  | **Total** | 31 (50.8%) |
| Adjuvant CTx, n (%) | EP | **9 (14.8%)** |
|  | IA/EP | 2 (3.3%) |
|  | ddMVAC | 2 (3.3%) |
|  | IO | 1 (1.6%) |
|  | Other**^†^** | 1 (1.6%) |
|  | **Total** | 15 (24.6%) |

**^*^** Other types of surgical resection include bilateral nephrouretectomy, left ureterectomy, and right nephroureterectomy.

**^†^** Other regimens of neoadjuvant chemotherapy include TMP, MVAC followed by ITP, irinotecan plus cisplatin, gemcitabine plus cisplatin plus taxotere, and single-agent cisplatin. Other regimens of adjuvant chemotherapy include ifosfamide plus adriamycin plus gemcitabine.

**SUPP. FIGURE 3**. Pie-in-pie chart showing the distributions of organs of relapse with biopsies, in **Cohort B** (**n=61**).

**SUPP. FIGURE 4.** Donut charts showing the distributions of pathologic patterns (inner donut) and the sub-distributions of co-existing pathologies with mixed SCUC, including predominant and minority SCUC (outer donut) at original diagnosis of SCNEC (**Panel A**) and at metastatic biopsy in **Cohort B** (**Panel B**). Sankey diagram showing the change of histologies of pathologic specimens at localized disease (LD [TUR]) and metastatic disease (MD), in **Cohort B (Panel C).** Distribution of histologies at MD per histologies at LD (**Panel D**).


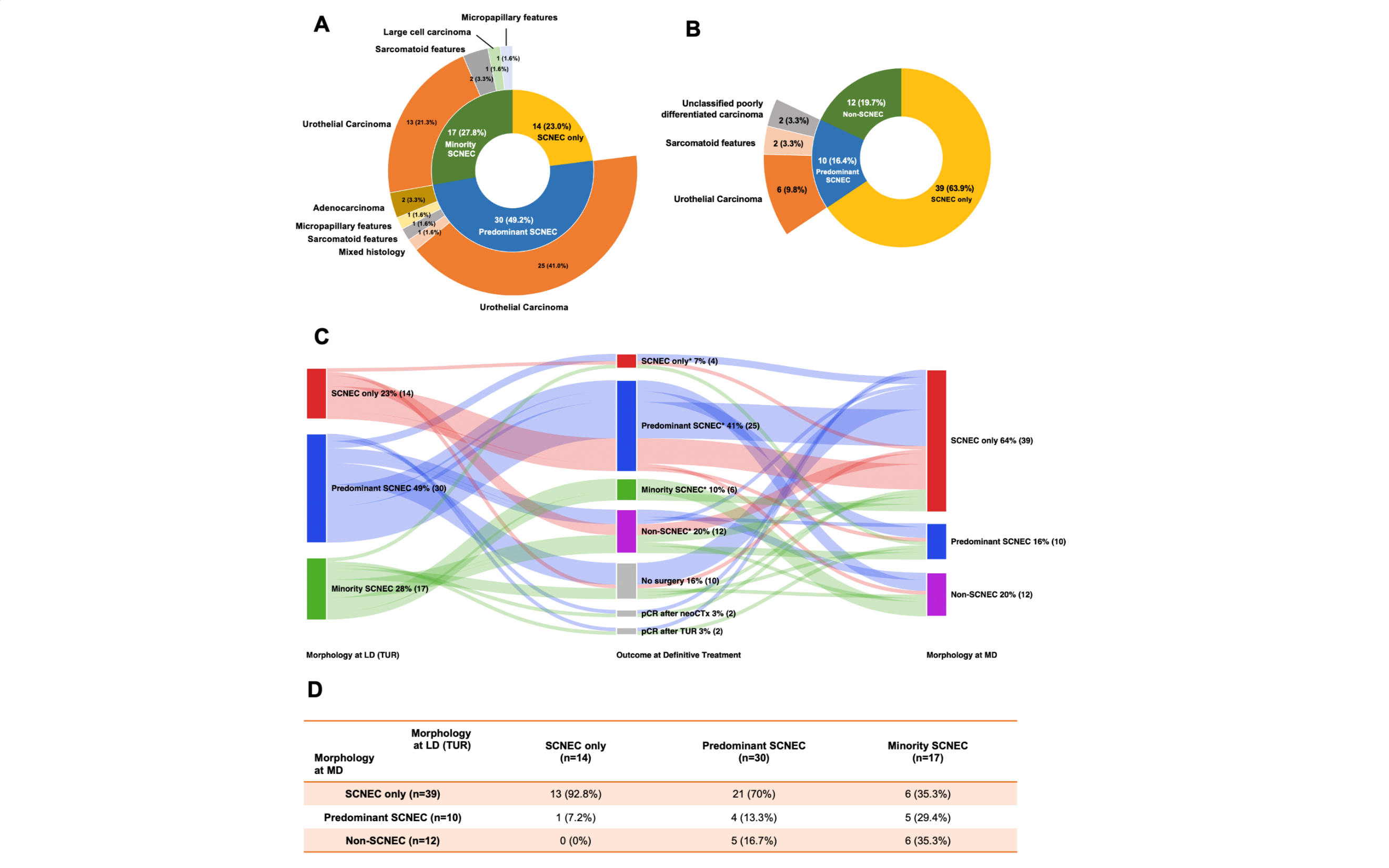


*Histologies of biospecimens from patients with surgical resection of primary.

LD: Localized disease; MD: Metastatic disease; SCNEC: Small cell neuroendocrine carcinoma; neoCTx: neoadjuvant chemotherapy; TUR: transurethral resection.

**SUPP. TABLE 4**. Distribution of visceral metastasis, with organ breakdown, and nodal-only metastasis, by histological subtype at metastatic-site biopsy in **Cohort B (n=61).**

|  | | SCNEC Only  (n=39) | Predominant SCNEC  (n=10) | Non-SCNEC  (n=12) | *P* value | All  (n=61) |
| --- | --- | --- | --- | --- | --- | --- |
| Visceral Metastasis, n (%) | **Any** | 34 (87.2%) | 8 (80%) | 9 (75%) | 0.58 | 50 (82%) |
|  | **Lung** | 10 (25.6%) | 2 (20%) | 3 (25%) | 0.93 | 15 (24.6%) |
|  | **Liver** | 12 (30.8%) | 2 (20%) | 3 (25%) | 0.77 | 17 (27.9%) |
|  | **Brain** | 10 (25.6%) | 5 (50%) | 1 (8.3%) | 0.09 | 16 (26.2%) |
|  | **Bone** | 10 (25.6%) | 2 (20%) | 2 (16.7%) | 0.79 | 14 (23%) |
| Nodal-only metastasis, n (%) | | 5 (12.8%) | 2 (20%) | 3 (25%) | 0. | 11 (18%) |

SCNEC: Small cell neuroendocrine carcinoma.

**SUPP. FIGURE 5**. Kaplan-Meier survival of Cohort B (n=61), per histology at metastatic-site biopsy. *Abbreviations*: SCNEC: Small cell neuroendocrine carcinoma, OS: Overall survival.
